# Threesomes destabilise certain relationships: multispecies interactions between wood decay fungi in natural resources

**DOI:** 10.1093/femsec/fix014

**Published:** 2017-02-08

**Authors:** Jennifer Hiscox, Melanie Savoury, Selin Toledo, James Kingscott-Edmunds, Aimee Bettridge, Nasra Al Waili, Lynne Boddy

**Affiliations:** School of Biosciences, Cardiff University, Cardiff CF10 3AX, UK

**Keywords:** antagonism, community dynamics, decomposer interactions, intransitivity, multispecies interactions, wood decay fungi

## Abstract

Understanding interspecific interactions is key to explaining and modelling community development and associated ecosystem function. Most interactions research has focused on pairwise combinations, overlooking the complexity of multispecies communities. This study investigated three-way interactions between saprotrophic fungi in wood and across soil, and indicated that pairwise combinations are often inaccurate predictors of the outcomes of multispecies competition in wood block interactions. This inconsistency was especially true of intransitive combinations, resulting in increased species coexistence within the resource. Furthermore, the addition of a third competitor frequently destabilised the otherwise consistent outcomes of pairwise combinations in wood blocks, which occasionally resulted in altered resource decomposition rates, depending on the relative decay abilities of the species involved. Conversely, interaction outcomes in soil microcosms were unaffected by the presence of a third combatant. Multispecies interactions promoted species diversity within natural resources, and made community dynamics less consistent than could be predicted from pairwise interaction studies.

## Teaser

Multispecies interactions between wood decay fungi promote species diversity within natural resources, and make community dynamics less consistent than could be predicted from pairwise interaction studies.

## INTRODUCTION

Understanding the forces that structure ecological communities is crucial for determining the contribution of these communities to ecosystem processes and their resilience to environmental change. Community dynamics are largely determined by interactions between individuals, making studying interactions key to explaining community development. However, whilst much research has been undertaken into characterising the mechanisms and outcomes of pairwise interactions, real-world communities comprise multiple species interacting simultaneously, and the complexity of these communities escalates with the addition of species, as the number of pairwise interconnections between species grows non-linearly (Beckage, Gross and Kauffman 2011). Models of plant and phytoplankton community development have shown that pairwise interactions are poor predictors of the progress and outcomes of these multispecies interactions (Huisman and Weissing 2001; Weigelt *et al.*^2007^).

Wood decay fungi are ideal for studying interactions between multiple competitors; they are easy to culture, and during competition for territory within woody resources they remain distinct (no merging) which makes patterns of territory gain or loss easy to determine. Antagonistic mycelial interactions (interference competition) are key to wood decay fungal ecology, occurring where there is an overlap in the niches of different species or strains (Woodward and Boddy 2008). Interactions may result in deadlock, where neither competitor gains any territory from the other, or replacement of one mycelium by another, although a spectrum of outcomes can occur between these extremes (Boddy 2000). Antagonism, thus, determines community development, and hence decay rate and carbon turnover in forest ecosystems. Antagonism is mediated through energetically expensive morphological and metabolic changes in competing mycelia, such as the production of barrages and invasive cords, and the secretion of antifungal toxins, metabolites and oxidative enzymes (Boddy and Heilmann-Clausen 2008; Hiscox *et al.*2015b).

Wood decay fungi can be roughly grouped by combative ability, which is broadly related to the timing of occurrence of the species in the successional community within woody resources and their life-history strategy (Rayner and Boddy 1988). Primary colonisers of dead woody resources, which may have been latently present in the living tree or arrive at the resource as spores (Parfitt *et al.*^2010^), rapidly proliferate through the uncolonised wood, competing with other developing mycelia for territory (R- or RS-selected; Boddy and Hiscox 2016). Primary colonisers are replaced by early secondary colonisers (C-selected), which typically arrive at the resource as spores. Early secondary colonisers are in turn replaced by later secondary or end-stage colonisers, which arrive as spores or as mycelial cords, which are linear aggregations of hyphae that grow out of colonised resources, foraging for new ones (Fricker and Bebber 2008). There exists a general hierarchy of combative ability where primary colonisers are the least combative and late secondary colonisers are the most, but intransitive (non-hierarchical) relationships are common between wood decay fungi (Boddy 2000; Laird and Schamp 2006). The simplest example of intransitive competition is the game of rock–paper–scissors, in which rock is covered by paper, paper is cut by scissors and scissors are crushed by rock (Rock>Paper>Scissors>Rock; Laird 2014). Furthermore, whilst certain species are not combative, they may be able to outcompete other species in some situations due to their tolerance of specific environmental stresses (S-selected; Boddy and Heilmann-Clausen 2008).

Though several simple studies of antagonistic interactions involving several wood decay fungi have been performed (Schoeman, Webber and Dickinson 1996; Boddy and Abdalla 1998; White *et al.*^1998^; Sturrock *et al.*^2002^; A’Bear *et al*. 2013; Toledo *et al.*^2016^), none have assessed interactions within woody resources and their effects on decomposition. Recently, three-way interactions between fungi in agar culture revealed different outcomes compared to pairwise interactions, with spatial positioning of the three competitors affecting their combative success (Toledo *et al.*^2016^). However, outcomes of interactions in artificial media often do not reflect well the dynamics in natural substrata, as nutrient and other environmental conditions are so different (Watkinson *et al.*^2006^).

The aim of this study was to test the following hypotheses in three-way interactions in wood and across soil: (1) the presence of a third combatant alters interaction outcomes compared to the results of separate pairwise interactions; (2) the relative position of each species affects interaction outcomes; (3) altered interaction outcomes affect resource decomposition rates.

## MATERIALS AND METHODS

### Preparation of inocula

Six native, beech (*Fagus sylvatica*) inhabiting basidiomycetes, comprising species with different ecological roles (primary coloniser: *Vuilleminia comedens*; early secondary colonisers: *Trametes versicolor, Stereum hirsutum, Bjerkandera adusta*; late secondary colonisers: *Hypholoma fasciculare, Phanerochaete velutina*; Table S1, Supporting Information), were maintained on 0.5% malt agar (MA: 15 g l^−1^ agar, 5 g l^−1^ malt extract; Lab M, UK). Beech wood blocks (2×2×1 cm) were sterilised by autoclaving three times over 72 h. Inocula were prepared by incubating wood blocks on colonised MA at 20°C in the dark for 12 weeks. The initial decay state of blocks was determined by destructively measuring dry weight/fresh volume (10 replicates/fungal species).

### Experimental treatments

Three experimental treatments were established. (1) The effect of a non-cord-forming species (*V. comedens, T. versicolor, S. hirsutum* or *B. adusta*) on the interaction between two cord formers (*P. velutina* and *H. fasciculare*) was determined. For each combination, species were positioned either with *H. fasciculare* in the middle, *P. velutina* in the middle or the non-cord-forming species in the middle. (2) The effect of proximity to extra uncolonised resources on interactions between two species was determined. A sterilised uncolonised wood block was used as the extra resource, and interactions were set up such that either one of the two species was adjacent to the extra resource, or it was positioned between them. (3) The effect of relative position on interactions between the non-cord-forming secondary coloniser species (*T. versicolor, S. hirsutum* and *B. adusta*) was determined, with species positioned in all combinations.

### Establishment of interactions in wood blocks

**Figure 1.**
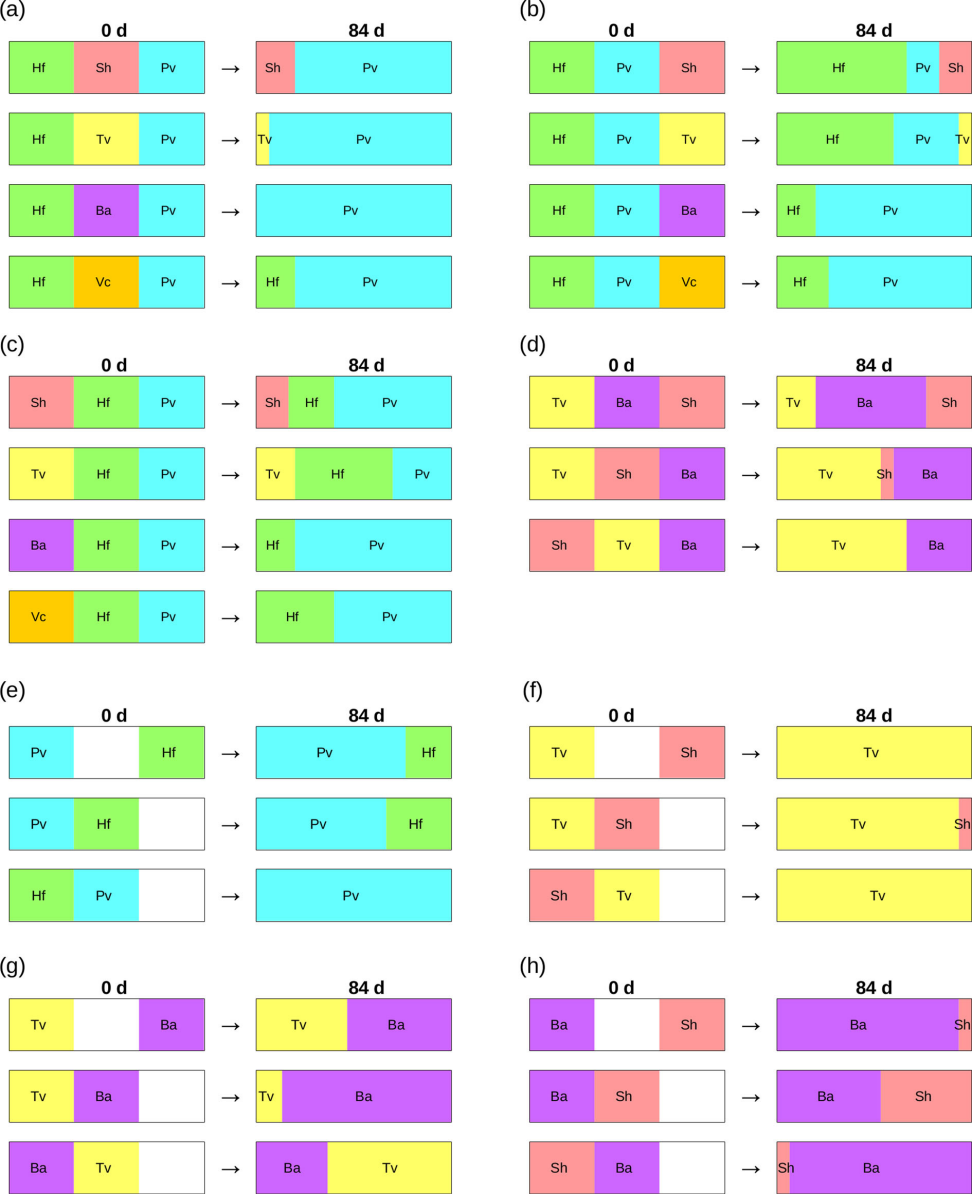
Representation of territory changes during interactions between three species in wood blocks (**a–d**), and between two species in wood blocks, where one or both species were adjacent to extra resources (i.e. an uncolonised block) (**e–h**). (**a**) Interactions where *H. fasciculare* (Hf) and *P. velutina* (Pv) flanked a non-cord-forming species (either *S. hirsutum*, *T. versicolor*, *B. adusta* or *V. comedens*: Sh, Tv, Ba and Vc, respectively); (**b**) interactions where Pv was positioned between Hf and a non-cord-forming species; (**c**) interactions where Hf was positioned between Pv and a non-cord-forming species; (**d**) interactions between the non-cord-forming species Tv, Ba and Sh in all combinations; (**e**) interactions between Hf and Pv; (**f**) interactions between Sh and Tv; (**g**) interactions between Tv and Ba; (**h**) interactions between Ba and Sh. Each species occupied an equal portion of territory at 0 day (left column). Relative sizes of territory at harvest (84 days; right column) are shown, based on extent of replacement (partial or complete), averaged across five replicates per interaction combination. White squares indicate the position of the blank territory.

Precolonised blocks were scraped free of adhering mycelium; three blocks precolonised with different species were arranged into interactions, with the cut vessels touching so the grain ran in the same orientation (continuously) in all blocks (see Fig. 1, left-hand columns). Blocks were held together with a sterile rubber band which was removed after 5 days, and incubated in 100 ml lidded plastic deli pots (Cater4you, UK) containing 30 ml sterile perlite moistened to –0.012 MPa (determined by the method of Fawcett and Collins-George 1967) in the dark at 20°C. A hole in the pot wall (∼2 mm diameter) covered in microporous surgical tape (3 M, UK) allowed aeration, and pots were watered fortnightly to maintain water potential. Five replicate interactions were set up for each combination.

### Establishment of interactions across soil

Soil was collected from mixed deciduous woodland (Coed Beddick, Tintern, UK) to 20 cm depth, air-dried, sieved through a 2 mm mesh and then frozen overnight at –20°C to kill any soil microfauna. The soil matric potential was adjusted to –0.012 MPa, and 200 g evenly compacted into square (24 × 24 cm) bioassay trays (Nunc-Gibco, UK). Colonised blocks were scraped free of adhering mycelium and placed on the soil surface; three blocks were placed on each tray, with the middle block positioned in the exact centre of the tray, and the other two evenly spaced on either side. Timing of block addition was staggered to ensure that both cord formers were of a similar size when they met: *H. fasciculare* blocks were added 2 days prior to *P. velutina* blocks. Non-cord-former blocks were added to the trays when the mycelium of an adjacent cord-former mycelium had grown to <1 cm away from the intended location of the non-cord-former block. Trays were incubated at 20°C in the dark, and watered (to maintain their starting water potential) and photographed every 7 days.

### Determining interaction outcomes

Outcomes were determined after 84 days for wood block interactions, and 42 days for soil tray interactions. The proportion of the three blocks colonised by each of the initial competitors was determined by reisolation. Blocks were separated and split in half along the grain using a sterile chisel. Pieces of wood (∼2 mm^3^) were excised from four regions within each block, representing each of 25% intervals progressing inward from the edge adjacent to the competitor block, inoculated onto 2% MA and incubated at 20°C until mycelium had emerged and could be identified morphologically. Interaction outcomes were classed as partial or complete replacement, depending on the extent of displacement of one combatant by another; or deadlock, where neither combatant gained or lost territory. Final density was determined from the half of the block not used for reisolation as previously described.

Outcomes were scored by calculating the percentage of the three blocks (combined) colonised by each coloniser at the end of the experiment: 100% indicates complete replacement of both competitors by the focal species (i.e. the focal species was recovered from every reisolation point), and 0% indicates complete replacement of the focal species by one or both competitors (i.e. the focal species was not recovered from any reisolation point). Scores were averaged over the five replicates performed for each combination.

### Image and statistical analysis

Images of soil microcosms were processed using ImageJ (NIH, USA). Following manual thresholding to remove soil from the images, hyphal coverage (cm^2^) was determined as the number of white pixels in a binary image. Mass fractal dimension (a measure of the space-filling capacity of a mycelium) was calculated using the box-counting method in the ImageJ FracLac plugin (Donnelly and Boddy 1998; Karperien 2013). Statistical analyses were performed using R (R Development Core Team 2014), and graphs generated using the R package ggplot2 (Wickham 2009). Block density losses were compared between different treatments using one-way ANOVA; data were checked for homogeneity of variances between groups using a Bartlett test prior to ANOVA, and post hoc diagnostic tests were performed to check normality of residuals within each group. Differences in % territory occupied by different focal species during three-way wood block interactions were compared using pairwise Mann-Whitney–Wilcoxon tests; data were not normally distributed (following a Shapiro-Wilk test); hence, a non-parametric test was used. Changes in hyphal coverage and fractal dimension over time between different interaction treatments were compared using a linear mixed-effects (lme) model with Tukey-Kramer *a posteriori* comparisons using the R package multcomp (Hothorn, Bretz and Westfall 2008), where effects of repeated measurements were controlled for by using biological replicate as a random effect. The lme compares differences between treatments, differences between timepoints, and whether there is an interaction between treatments and time points. Within these tests, treatment was considered to be the non-cord-forming species (or blank) interacting with both cord formers across soil. Prior to running the lme models, data were checked for normality within each treatment using a qqmath test, and a log transformation performed. Post hoc tests were also performed to check normality and variance of residuals (between treatments and within groups, respectively), and the normality of random effects was also checked.

## RESULTS

### Wood block interactions: effect of relative position on outcome

**Figure 2.**
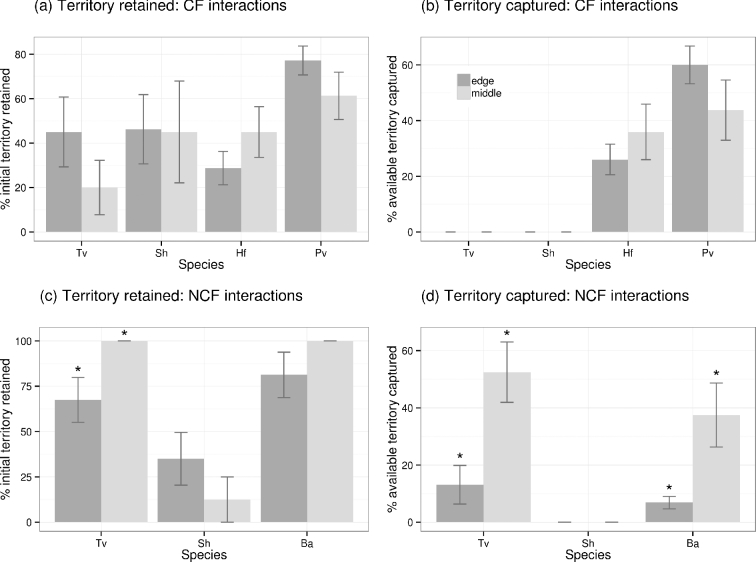
Differences in territory occupied by different focal species during three-way wood block interactions where species were positioned on the edge vs. positioned in the middle. Territory is calculated as the % occupation of the three wood blocks per interaction as determined by reisolation, and is averaged over all interaction combinations involving that species. Sh: *S. hirsutum*; Tv: *T. versicolor*; Ba: *B. adusta*; Hf: *H. fasciculare*; Pv: *P. velutina*. *Vuilleminia comedens* and Ba are omitted from A and B because they did not retain or capture any territory during these interaction combinations. Bars show the mean territory occupied ± standard error. Asterisk indicates a significant (*P* < 0.05) difference in territory occupation between treatments.

The effect of relative position of a fungus within an interaction was species and combination specific. During interactions with cord formers, both *Vuilleminia comedens* and *Bjerkandera adusta* were completely replaced by the cord formers (Figs 1 and 2a, b). Neither *Trametes versicolor* nor *Stereum hirsutum* captured any territory from the cord formers (Fig. 2a and b). No significant (*P* > 0.05) differences in territory retained or captured were detected when species were positioned on the edge vs. in between two combatants (Fig. 2a and b). During interactions between non-cord formers, neither *T. versicolor* nor *B. adusta* lost any territory when positioned in between two combatants, and both captured significantly (*P* < 0.05) more territory in this position (Figs 1 and 2c, d). *Stereum hirsutum* retained more territory when positioned on the edge during non-cord-former interactions, although this was not significant (*P* > 0.05), but did not capture any territory from *T. versicolor* or *B. adusta* in either position (Fig. 2c and d).

The consistency of outcomes between replicates varied within different treatments (Table 1). Interactions between non-cord formers had highly consistent outcomes, and although in many cases replicates differed between replacement and partial replacement, the species responsible for the replacement was consistent (Table 1C). Similarly, outcomes were highly consistent during replicate interactions where a non-cord former was positioned between the two cord formers; in these combinations, *Phanerochaete velutina* was always combatively dominant, at least partially replacing both competitors (Table 1A). However, when cord formers were positioned adjacent to each other, outcomes were highly inconsistent. For example, during interactions where *Hypholoma fasciculare* was positioned between *P. velutina* and *V. comedens, H. fasciculare* completely replaced *P. velutina* and *V. comedens* in two replicates, whilst in the other three replicates *P. velutina* replaced both of the other competitors (Table 1A).

**Table 1.** Outcomes of multispecies interactions in beech wood blocks.

**A.** Effect of a non-cord-forming species on the			**B.** Effect of proximity to extra resources			**C.** Interactions between non-cord-	
outcome of interactions between *P. velutina*			on interaction outcomes			forming secondary colonisers	
and *H. fasciculare*							
Interaction	NCF outcome	CF outcome	Interaction	NCF outcome	CF outcome	Interaction	NCF outcome
Pv-Sh-Hf	PR by Pv(3)	R by Pv	Pv-<>-Hf		R by Pv (3)	Tv-Ba-Sh	R/PR of Tv by Ba (5)
	D(2)				PR by Hf (1)		R/PR of Sh by Ba (3)
Pv-Tv-Hf	R by Pv(3)	R by Pv	Pv-Hf-<>		R by Pv (2)		D between Sh and Ba (2)
	PR by Pv(2)				R by Hf (1)	Sh-Tv-Ba	R of Sh by Tv (5)
Pv-Ba-Hf	R by Pv	R by Pv			PR by Hf (1)		PR of Tv by Ba (4)
Pv-Vc-Hf	R by Pv(3)	R by Pv (3)			PR by Pv (1)		PR of Ba by Tv (1)
	R by Hf(2)	D (2)	Hf-Pv-<>		R by Pv	Ba-Sh-Tv	R of Sh by Tv and Ba (4)
Hf-Pv-Sh	R by Hf(2)	R by Hf (4)	Tv-<>-Sh	R by Tv			PR of Sh by Ba (1)
	PR by Pv(2)	R by Pv (1)	Tv-Sh-<>	R by Tv (4)			
	D(1)			PR by Tv (1)			
Hf-Pv-Tv	R by Hf(2)	PR by Hf(3)	Sh-Tv-<>	R by Tv			
	R by Pv(2)	R by Hf(2)	Tv-<>-Ba	PR by Ba (3)			
	D(1)			PR by Tv (1)			
Hf-Pv-Ba	R by Pv (4)	R by Pv (4)		D (1)			
	R by Hf (1)	R by Hf (1)	Tv-Ba-<>	R by Ba (3)			
Hf-Pv-Vc	R by Pv	R by Pv (3)		D (2)			
		PR by Hf (2)	Ba-Tv-<>	D (4)			
Pv-Hf-Sh	R by Pv(2)	R by Pv (4)		PR by Tv (1)			
	D(2)	R by Hf (1)	Ba-<>-Sh	R of Sh by Ba (4)			
	R by Hf(1)			D (1)			
Pv-Hf-Tv	D(3)	R by Pv (2)	Ba-Sh-<>	PR of Sh by Ba			
	PR by Hf(1)	R by Hf (2)	Sh-Ba-<>	R by Ba (4)			
	R by Hf(1)	PR by Hf (1)		D (1)			
Pv-Hf-Ba	R by Hf(3)	R by Pv (2)					
	R by Pv(2)	PR by Hf (1)					
		R by Hf (1)					
Pv-Hf-Vc	R by Pv(3)	R by Pv (3)					
	R by Hf(2)	R by Hf (2)					

NCF outcome: outcome of interactions occurring in block originally colonised by a non-cord former, or an uncolonised block. CF outcome: outcome of interactions between the cord-forming fungi *P. velutina* and *H. fasciculare*. R: replaced; PR, partially replaced; D: deadlock. Number of replicates resulting in each outcome is given in parentheses, where no number is given all five replicates resulted in the same outcome.

None of the non-cord-forming species captured any territory from cord formers during interactions; *B. adusta* and *V. comedens* were completely replaced by the cord formers in all interactions, and *S. hirsutum* and *T. versicolor* were at least partially replaced (Table 1A; Fig. 1a–c). Including *V. comedens* as the third competitor in the interaction between *H. fasciculare* and *P. velutina* resulted in similar outcomes to interactions involving an uncolonised extra resource (Table 1A and B; Fig. 1a–c, e). In contrast, including *B. adusta* as a third competitor increased the overall combative success of *P. velutina*, although the increase was consistent only during interactions where *B. adusta* was positioned in the middle (Table 1A; Fig. 1a–c). Including *T. versicolor* or *S. hirsutum* as the third competitor improved *P. velutina* combative success when positioned in the middle, but improved *H. fasciculare* combative success when positioned on the edge adjacent to either competitor (Table 1A; Fig. 1a–c).

During interactions between non-cord formers, *T. versicolor* was most combatively successful when positioned next to *S. hirsutum*, which was at least partially replaced by *T. versicolor* (Fig. 1d). *Bjerkandera adusta* did not lose any territory during non-cord former interactions, but was most successful when positioned in the middle, capturing territory from both *S. hirsutum* and *T. versicolor* (Fig. 1d).

### Wood block interactions: effect of access to extra resources

**Figure 3.**
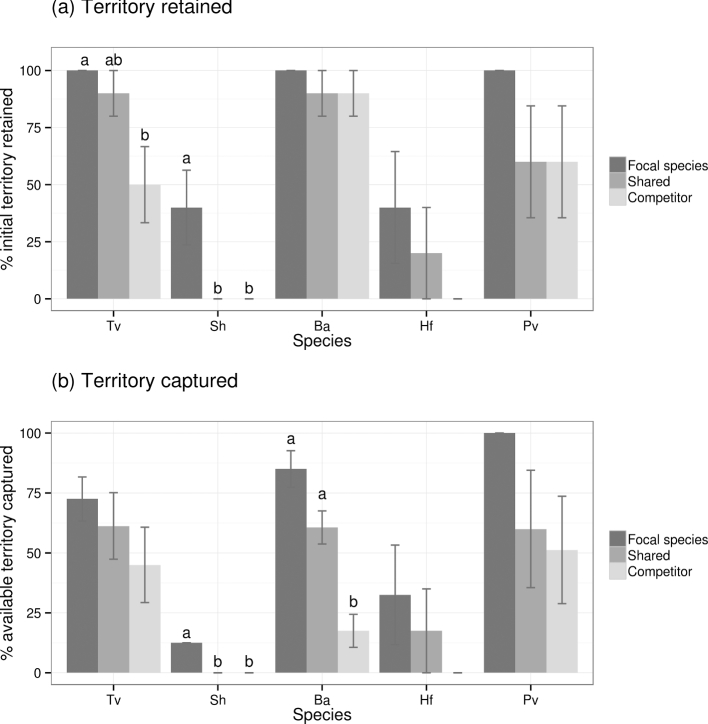
Differences in territory occupied by different focal species during three-way wood block interactions involving extra resources. Extra resources were possessed either by the focal species, or were shared, or were possessed by the competitor. Territory is calculated as the % occupation of the three wood blocks per interaction as determined by reisolation; bars indicate the mean territory occupied ± standard error and (averaged over all interaction combinations involving that species). Sh: *S. hirsutum*; Tv: *T. versicolor*; Ba: *B. adusta*; Hf: *H. fasciculare*; Pv: *P. velutina*. Different letters indicate significant (*P* < 0.05) differences in territory occupation between treatments.

Overall, all species were most successful, in terms of territory retention and capture, when in sole possession of extra resources, and least successful when the resource was possessed by the competitor, although these differences were not always significant (Figs 1 and 3; *P* > 0.05). The location of extra resources had no significant effect (*P* > 0.05) on the territory retained or captured by *H. fasciculare* and *P. velutina* (Fig. 3). *Stereum hirsutum* was completely replaced when the competitor had sole or shared access to the extra resource (Fig. 3). *Trametes versicolor* was significantly (*P* < 0.05) better able to retain territory when it had access to extra resources, even if they were shared, but this had no significant (*P* > 0.05) effect on its ability to capture territory (Fig. 3). Conversely, *B. adusta* was significantly (*P* < 0.05) better able to capture territory when it had access to extra resources, even if they were shared, but this had no significant (*P* > 0.05) effect on its ability to retain territory (Fig. 3). Where the weaker competitor of the pairing was positioned between the extra resource and the other fungus, the former was often displaced into the extra resource as the stronger competitor captured territory in the block originally colonised by the weaker competitor: for example, during interactions between *T. versicolor* and *S. hirsutum*, the weaker competitor (*S. hirsutum*) was only detected in the distant edge of the extra resource, having been displaced from the rest of the potential territory by *T. versicolor* (Fig. 1f).

### Wood block interactions: effects on decomposition

No significant (*P* > 0.05) differences in wood block density were detected between interaction combinations, with two exceptions. During pairings between *T. versicolor* and *B. adusta*, total weight loss was significantly (*P* < 0.05) lower if *B. adusta* was adjacent to the extra resource than if *T. versicolor* was adjacent to the extra resource (Table S2, Supporting Information). Furthermore, during pairings between *H. fasciculare*, *P. velutina* and *S. hirsutum*, total weight loss was significantly (*P* < 0.05) higher when *H. fasciculare* was positioned in the middle than if *P. velutina* was positioned in the middle (Table S2).

### Soil tray interactions: effect of relative position on outcome

**Figure 4.**
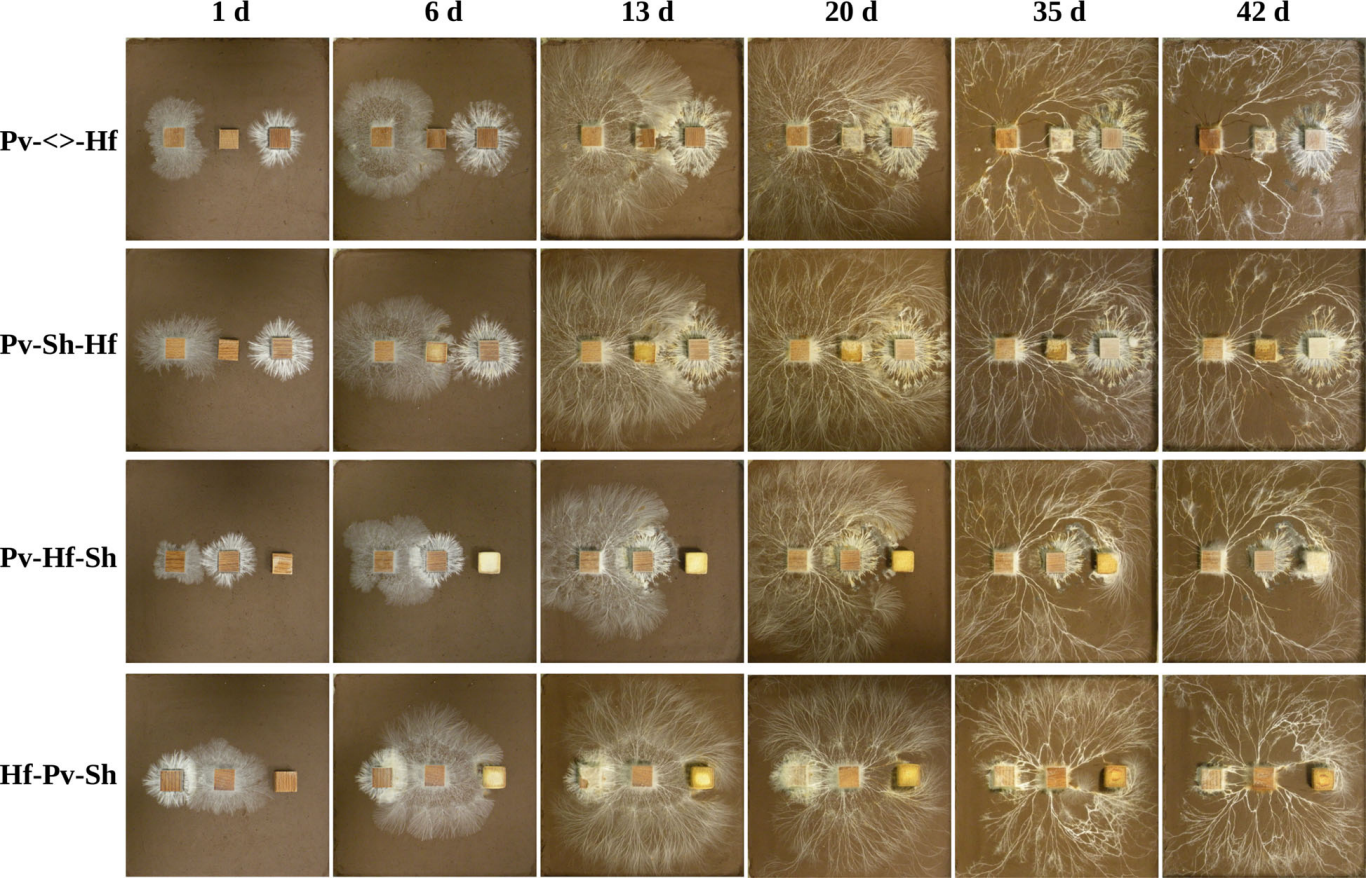
Mycelial cord system development over compacted soil, in 24 × 24 cm trays, during multispecies interactions over 42 days. Top row: *P. velutina* (Pv; left) and *H. fasciculare* (Hf; right) share an extra resource (<>; middle). Second row: The non-cord-forming fungus *S. hirsutum* (Sh) is positioned between Pv (left) and Hf (right). Third row: Hf is positioned between Pv (left) and Sh (right). Bottom row: Pv is positioned between Hf (left) and Sh (right).

Relative position of the competitors had little effect on soil tray interaction outcomes, and, unlike wood block interactions, the position of the blocks initially colonised by cord formers did not affect the consistency of outcomes between replicates (Table S3, Supporting Information). Visual interpretation showed that in all combinations *P. velutina* mycelium had at least partially overgrown competitor wood blocks by 42 days (Fig. 4; Fig. S1a–d, Supporting Information); however, overgrowth and replacement of one mycelium by another cannot be distinguished between using visual methods alone. Unfortunately, reisolation success was impaired due to high rates of contamination, resulting in low replicate numbers for some combinations (Table S3).

### Soil tray interactions: effect on hyphal coverage and fractal dimension

Total hyphal coverage of cord systems of interacting *H. fasciculare* and *P. velutina* increased up to 20 days, decreased by 35 days and then either stayed the same or slightly increased by 42 days (Table 2; Fig. 4). This pattern was consistent between interactions involving different non-cord-forming competitors, and during interactions with competitors in different relative positions. Interactions involving *B. adusta* or *V. comedens* had significantly higher (*P* < 0.05) total hyphal coverage at 20 days than other combinations, but at all other timepoints no significant differences (*P* > 0.05) were detected (Table 2; Fig. S2, Supporting Information). Fractal dimension of cord systems increased between 1 and 20 days, and then remained constant until 42 days (Table 2). The identity of the non-cord-forming competitor had no significant (*P* > 0.05) effect on cord system fractal dimension, and neither did the presence of an uncolonised resource (Table 2; Fig. S3, Supporting Information).

**Table 2.** Hyphal coverage and mass fractal dimension of cord systems during multispecies interactions.

	Hyphal coverage cm^2^ (mean ± SEM)				Fractal dimension D_BM_ (mean ± SEM)			
Interaction	1 day	20 days	35 days	42 days	1 day	20 days	35 days	42 days
Hf-Pv-<>	35.05 ± 4.44	111.91 ± 18.74	64.10 ± 3.25	86.11 ± 5.76	1.669 ± 0.011	1.768 ± 0.014	1.762 ± 0.011	1.747 ± 0.010
Pv-Hf-<>	27.54 ± 7.90	85.96 ± 6.82	54.51 ± 5.54	53.40 ± 8.11	1.584 ± 0.083	1.742 ± 0.004	1.744 ± 0.012	1.681 ± 0.024
Pv-<>-Hf	32.22 ± 3.56	108.77 ± 17.23	57.54 ± 3.89	72.20 ± 5.79	1.640 ± 0.022	1.747 ± 0.023	1.745 ± 0.010	1.711 ± 0.015
Hf-Pv-Vc	46.93 ± 2.84	171.30 ± 33.81 ab	59.90 ± 5.36	102.60 ± 8.09	1.692 ± 0.005	1.787 ± 0.020	1.754 ± 0.019	1.749 ± 0.009
Pv-Hf-Vc	47.02 ± 4.23	120.66 ± 15.20 a	47.19 ± 3.84	73.36 ± 8.60	1.692 ± 0.017	1.767 ± 0.008	1.730 ± 0.011	1.714 ± 0.017
Pv-Vc-Hf	46.07 ± 3.90	199.93 ± 18.78 b	54.65 ± 6.48	71.73 ± 7.43	1.718 ± 0.006	1.798 ± 0.009	1.747 ± 0.007	1.702 ± 0.019
Hf-Pv-Tv	42.18 ± 5.58	87.67 ± 8.16 a	46.69 ± 8.78	67.11 ± 7.45 a	1.700 ± 0.011	1.735 ± 0.008	1.702 ± 0.025 a	1.703 ± 0.017 a
Pv-Hf-Tv	36.70 ± 2.73	82.90 ± 7.70 ab	55.39 ± 5.72	66.12 ± 6.93 ab	1.668 ± 0.011	1.726 ± 0.020	1.748 ± 0.011 b	1.721 ± 0.015 b
Pv-Tv-Hf	46.08 ± 2.05	116.27 ± 12.96 b	61.21 ± 6.57	99.26 ± 10.97 b	1.679 ± 0.004	1.765 ± 0.009	1.740 ± 0.015 b	1.758 ± 0.015 b
Hf-Pv-Sh	42.18 ± 5.58	87.67 ± 8.16 a	46.69 ± 8.78	67.11 ± 7.45	1.700 ± 0.011	1.735 ± 0.008	1.702 ± 0.025	1.703 ± 0.017
Pv-Hf-Sh	26.19 ± 3.43	97.24 ± 12.40 b	47.57 ± 5.57	65.71 ± 10.47	1.604 ± 0.023	1.745 ± 0.011	1.718 ± 0.015	1.688 ± 0.030
Pv-Sh-Hf	47.03 ± 3.24	137.34 ± 11.08 b	49.35 ± 5.28	68.97 ± 2.63	1.673 ± 0.025	1.772 ± 0.019	1.711 ± 0.013	1.700 ± 0.005
Hf-Pv-Ba	29.82 ± 12.27	166.68 ± 14.24	50.30 ± 2.50	103.69 ± 12.27	1.631 ± 0.016	1.785 ± 0.008	1.743 ± 0.005	1.742 ± 0.013
Pv-Hf-Ba	41.85 ± 6.64	128.83 ± 10.65	37.89 ± 6.96	65.46 ± 8.88	1.664 ± 0.023	1.779 ± 0.007	1.706 ± 0.030	1.685 ± 0.025
Pv-Ba-Hf	36.29 ± 5.52	141.18 ± 46.36	47.39 ± 7.62	93.96 ± 18.50	1.629 ± 0.026	1.769 ± 0.028	1.702 ± 0.037	1.697 ± 0.033

Hf: *H. fasciculare*; Pv: *P. velutina*; Sh: *S. hirsutum*; Tv: *T. versicolor*; Ba: *B. adusta*; Vc: *V. comedens*), or an uncolonised block (<>). Significant (*P* < 0.05) differences between treatments (within each group of three) are indicated by different letters; no letters in a group indicates no significant (*P* > 0.05) differences.

Relative wood block position had no effect on total hyphal coverage where interactions involved *B. adusta* or an uncolonised wood block. Interactions where *V. comedens* was positioned in the middle (Pv-Vc-Hf) had significantly (*P* < 0.05) lower total hyphal coverage at 20 days compared to interactions involving the same species combination where *H. fasciculare* was positioned in the middle (Pv-Hf-Vc; Table 2). Similarly, interactions where *T. versicolor* was positioned in the middle (Pv-Tv-Hf) had significantly (*P* < 0.05) higher total hyphal coverage at 20 and 42 days compared to interactions where *P. velutina* was positioned in the middle (Hf-Pv-Tv; Table 2). Only during interactions where *S. hirsutum* was positioned in the middle (Pv-Sh-Hf) was total hyphal coverage significantly (*P* < 0.05) higher than both other interactions involving the same species combination at 20 days (Table 2).

Relative wood block position had no effect on fractal dimension of cord systems in interactions involving uncolonised wood blocks, *B. adusta*, *V. comedens* or *S. hirsutum* (Table 2). However, during interactions involving *T. versicolor*, combinations where *P. velutina* was positioned in the middle (Hf-Pv-Tv) had significantly (*P* < 0.05) lower fractal dimension at 35 and 42 days compared to both other interactions involving the same species combination (Table 2).

## DISCUSSION

Multispecies competition is a very complicated process, and because of this, most studies of interactions have focused on pairwise combinations. However, our data show that pairwise combinations are not always accurate predictors of the outcomes of multispecies competition between fungi in natural resources, similar to results of previous experiments with plants (Weigelt *et al.*^2007^) and macroecological models (Huisman and Weissing 2001; Laird 2014). Furthermore, outcomes of multispecies interactions in wood blocks were often less consistent than in pairwise combinations. For meaningful results to be generated to feed into community models, it is thus essential to perform experiments with multiple combatants.

Models of competition, based on those widely used in plant and phytoplankton ecology, in which multiple species compete for limiting resources (Tilman 1982), show that it is impossible to predict the winner of multispecies competition with certainty (Huisman and Weissing 2001). In part this is due to the impossibility of measuring initial conditions with sufficient precision, since the tiniest differences in initial conditions may lead to a different outcome of competition (Huisman and Weissing 2001). These models also show that winners of multispecies competition can be unpredictable even in a fully deterministic setting without any stochastic elements (Huisman and Weissing 2001). That said, species with appropriate traits still have a higher probability of dominating than species with inappropriate traits, as long as the species involved are each potentially strong enough to replace their competitors (Huisman and Weissing 2001). Our study shows that cord formers dominate during interactions with non-cord formers: no non-cord formers managed to capture any cord-former territory in either wood block or soil tray interactions. During pairwise interactions, albeit using different strains to this study, *Phanerochaete velutina* consistently replaced *Hypholoma fasciculare* (both cord formers; Hiscox *et al.*2015b); however, during multispecies wood block interactions complete reversals in outcomes between these cord formers were frequently observed between replicates of the same interaction. Reversals occurred independent of the identity of a third competitor. This variation in outcome among replicates of the same combination and spatial arrangement has also been observed in multispecies interactions in the less environmentally realistic setting of agar (Sturrock *et al.*^2002^; Toledo *et al.*^2016^). The presence of a third competitor might increase the likelihood of small differences in initial conditions, making outcomes less predictable as mentioned above. The reversals in outcomes during cord-former interactions are likely to impact community function, because although both *H. fasciculare* and *P. velutina* occupy the same ecological niche, they decompose wood at different rates (Worrall, Anagnost and Zabel 1997) and may have differing effects on subsequent community development (Hiscox *et al.*2015a, 2016).

Interestingly, *P. velutina* was much more dominant than *H. fasciculare* in soil microcosms, which exhibited no reversals in outcome (i.e. were more consistent) despite the presence of a third competitor. *Phanerochaete velutina* had greater combative success (retained or captured more territory) during multispecies interactions in wood and in soil microcosms than in agar (Toledo *et al.*^2016^), whereas the reverse was true for *H. fasciculare* and the non-cord formers (Toledo *et al.*^2016^). However, the lower combative ability of *P. velutina* on agar may be artificial, since interaction duration may have been too short (albeit 8 weeks) to allow the outcome to fully resolve (Toledo *et al.*^2016^), or that its strong decomposer ability gives it an advantage in natural resources, which are nutrient-poor resources relative to malt agar (Watkinson *et al.*^2006^). This impact of substrate on outcomes highlights the importance of using natural substrata during studies of community composition and development.

Excepting the primary coloniser *Vuilleminia comedens*, the non-cord-forming species used in this experiment have similar combative abilities: in pairwise combinations in wood blocks they all deadlock with each other (Hiscox *et al.*2015b). However, during multispecies interactions, or in the presence of extra resources, *Stereum hirsutum* was clearly less combative than either *Trametes versicolor* or *Bjerkandera adusta*. This reversal of interaction outcomes may indicate different combative strategies during different scenarios, and is likely to have a large impact on subsequent community development, since pre-colonisation of resources by *S. hirsutum* has been shown to lead to a successional community distinct from that following *T. versicolor* and *B. adusta* (Hiscox *et al.*2015a).

Not all multispecies interactions (i.e. cord formers against non-cord formers) were unpredictable from pairwise outcomes. Replacement of *B. adusta* and *V. comedens* by cord formers during multispecies interactions in wood blocks supported previous results from pairwise interactions (Hiscox *et al.*2015b); this was to be expected for the latter, which was the only early coloniser and relatively poor competitor in the current study. Similarly, patterns of fungal dominance evident in soil tray interactions between the cord formers *P. velutina, H. fasciculare* and *Resinicium bicolor* supported previous work employing pairwise interactions between the competitors in similar experimental systems (Crowther, Boddy and Jones 2011; A’Bear *et al*. 2013). Neither spatial position of combatants nor identity of the third combatant greatly affected mycelial cord system development in soil microcosms, in terms of hyphal coverage or fractal dimension (branching), despite differences in territory occupied by the cord formers during these interactions. However, the high level of contamination in reisolation cultures may have impacted the outcomes of interactions, possibly masking any variability caused by different treatments.

Certain combinations of the species used in the present paper showed a degree of intransitivity in outcomes during pairwise competition. For example, in wood block interactions *P. velutina* replaced *H. fasciculare, H. fasciculare* replaced *S. hirsutum*, but *P. velutina* deadlocked with *S. hirsutum* (Hiscox *et al.*2015b). Similarly, whilst *P. velutina* replaced *H. fasciculare* and *T. versicolor* in pairwise combinations, *T. versicolor* and *H. fasciculare* deadlocked (Hiscox *et al.*2015b). Although neither of these examples exhibit true intransitivity (which would have involved replacement of the *P. velutina* by *S. hirsutum*, or of *H. fasciculare* by *T. versicolor*, not deadlock), the competition structure is almost cyclical and theoretically should promote coexistence, because despite the intense competition between pairs of species, there is not a clear hierarchy of superiority at the community level (Laird 2014). Spatially explicit interactions, such as those between fungi inhabiting wood blocks, have been shown to enhance intransitivity-mediated coexistence in simulations (Laird and Schamp 2006). Enhanced coexistence is evident from our results, which showed increased persistence in the resource of *S. hirsutum* and *T. versicolor*, in both wood block and soil interactions, compared to combinations without intransitivity.

The relative position of competitors within an interaction affected outcomes in wood blocks but not soil trays. Furthermore, the effects of relative position were species and combination specific. Perhaps most interesting is the effect of relative position on consistency of outcomes in cord-former interactions: where cord formers were positioned adjacent to each other, outcomes were less consistent than when they were separated by a non-cord former. Differences in antagonistic strategy may confer these spatial ‘preferences’. For example, *S. hirsutum* is better at retaining territory than capturing it from an opponent (Hiscox *et al.*2015b), and this defensive ability appears to be equally strong independent of whether it was confronted with two opponents simultaneously (when positioned in the middle) or when fighting a single competitor (when positioned on the edge). Exposure to different competitors may induce different antagonistic mechanisms in a mycelium (El Ariebi *et al.*^2016^), and if this mycelium were exposed to these competitors simultaneously (i.e. positioned in the middle) it may produce a greater antagonistic arsenal than if confronted with either competitor singly, making it more combatively successful, as observed for *B. adusta* and *T. versicolor* during non-cord-former interactions.

Exclusive access to extra resources made a species more combatively successful during pairwise interactions in wood blocks, but only marginally more than if the extra resource was shared. It is well established that the volume of territory occupied by a mycelium affects its combative ability (Holmer and Stenlid 1993; Sturrock *et al.*^2002^; Song *et al.*^2015^), likely due to the increased access to nutrients and larger mycelial mass resulting in greater ability to induce or defend against antagonistic mechanisms. Access to extra resources in soil tray interactions had no effect on interaction outcome nor on cord system development, possibly because cords enable both competitors to gain access to the resource, even if they are not directly adjacent to it.

It is clear that the combination of species present in a resource affects the rate of decomposition of that resource, since different species have different decay abilities (Worrall, Anagnost and Zabel 1997). For the majority of combinations studied here, the relative position of species within an interaction did not have an effect on wood decomposition rates. However, due to the short nature of this experiment, this is not definitive. During pairings between *T. versicolor* and *B. adusta*, decomposition rate was directly related to the amount of territory occupied by *T. versicolor* during these interactions, since *T. versicolor* decomposes wood at a higher rate than *B. adusta* (Hiscox *et al.*^2016^). Similarly, the higher decomposition ability of *P. velutina* relative to *H. fasciculare* (Hiscox *et al.*^2016^) explains the increased decomposition observed during interactions involving *S. hirsutum* where *P. velutina* was dominant, compared to interactions where *H. fasciculare* was dominant. Clearly, the spatial configuration of a decay community, as well as the composition of that community, is highly important in determining resource decomposition rates.

## CONCLUSIONS

Multispecies interactions between wood decay fungi in natural resources are more complex than their pairwise interactions would suggest when occurring in wood blocks, but to a lesser extent in soil microcosms. Neither presence of a third combatant nor relative position of competitors nor access to extra resources, affected outcomes of interactions, cord system development or consistency of outcomes between replicates in soil microcosms. The presence of a third combatant altered interaction outcomes compared to the results of separate pairwise interactions in wood blocks (hypothesis 1), especially in combinations with intransitivity, which appeared to promote species coexistence within the resource. The relative position of combatants in wood block interactions altered outcomes involving non-cord formers but not cord formers (hypothesis 2), with *Trametes versicolor* and *Bjerkandera adusta* more combatively successful when positioned between two competitors vs. positioned on the edge. However, the relative position of the non-cord-forming competitor during interactions with *Hypholoma fasciculare* and *Phanerochaete velutina* destabilised the consistency of interaction outcomes between replicates, with greater inconsistency when the cord formers were positioned adjacent to each other. As has been shown previously, access to extra resources by one combatant during pairwise interactions improved the combative ability of that combatant, likely due to increased nutrient availability to support antagonistic mechanisms. Altered interaction outcomes, resulting from difference in spatial position of competitors, led to differences in resource decomposition rates in two combinations (hypothesis 3), indicating the importance of interactions in determining wood decay rates.

## Supplementary Material

Supplemental material Supplementary data are available at *FEMSEC* online.
