# Supplementary material for: Threesomes destabilise certain relationships: multispecies interactions between wood decay fungi in natural resources
Source: FEMS Microbiol Ecol. 2017 Feb 8;93(3):fix014. doi: 10.1093/femsec/fix014 (PMC5399798; doi:10.1093/femsec/fix014)
Supplement: Supplemental material — Supplementary data are available at FEMSEC online. [file fix014_supp.zip › Figure_S1-S3.pdf]

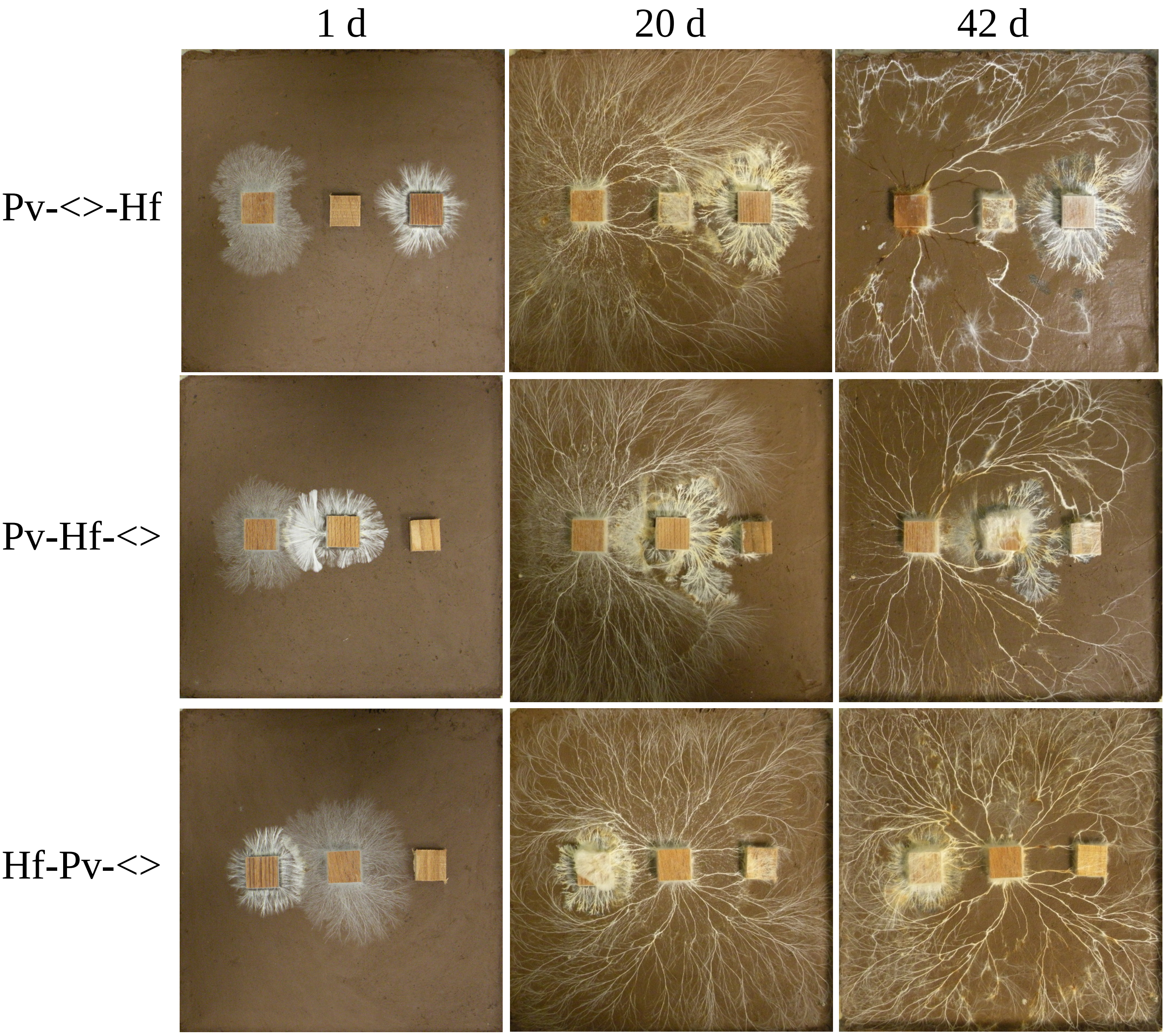

Supplementary Figure 1A. Progression of mycelial interactions between the cord-forming fungi *P. velutina* (Pv) and *H. fasciculare* (Hf) growing out of pre-colonised beech wood blocks, with uncolonised wood blocks (<>). Relative position of the blocks is assorted in all possible combinations.

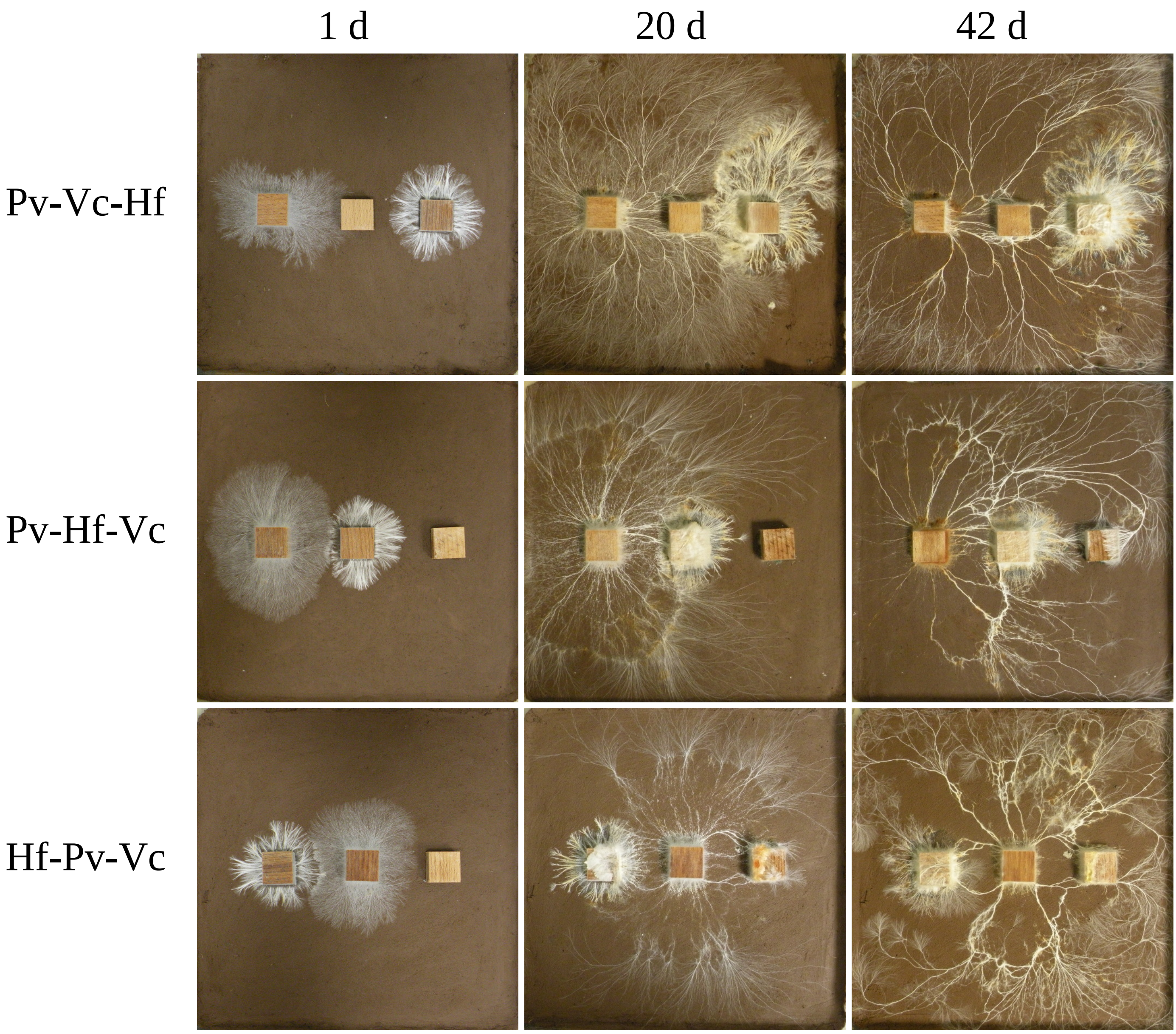

Supplementary Figure 1B. Progression of mycelial interactions between the cord-forming fungi *P. velutina* (Pv) and *H. fasciculare* (Hf) with the non-cord-forming fungus *V. comedens* (Vc), growing out of pre-colonised beech wood blocks. Relative position of the blocks is assorted in all possible combinations.

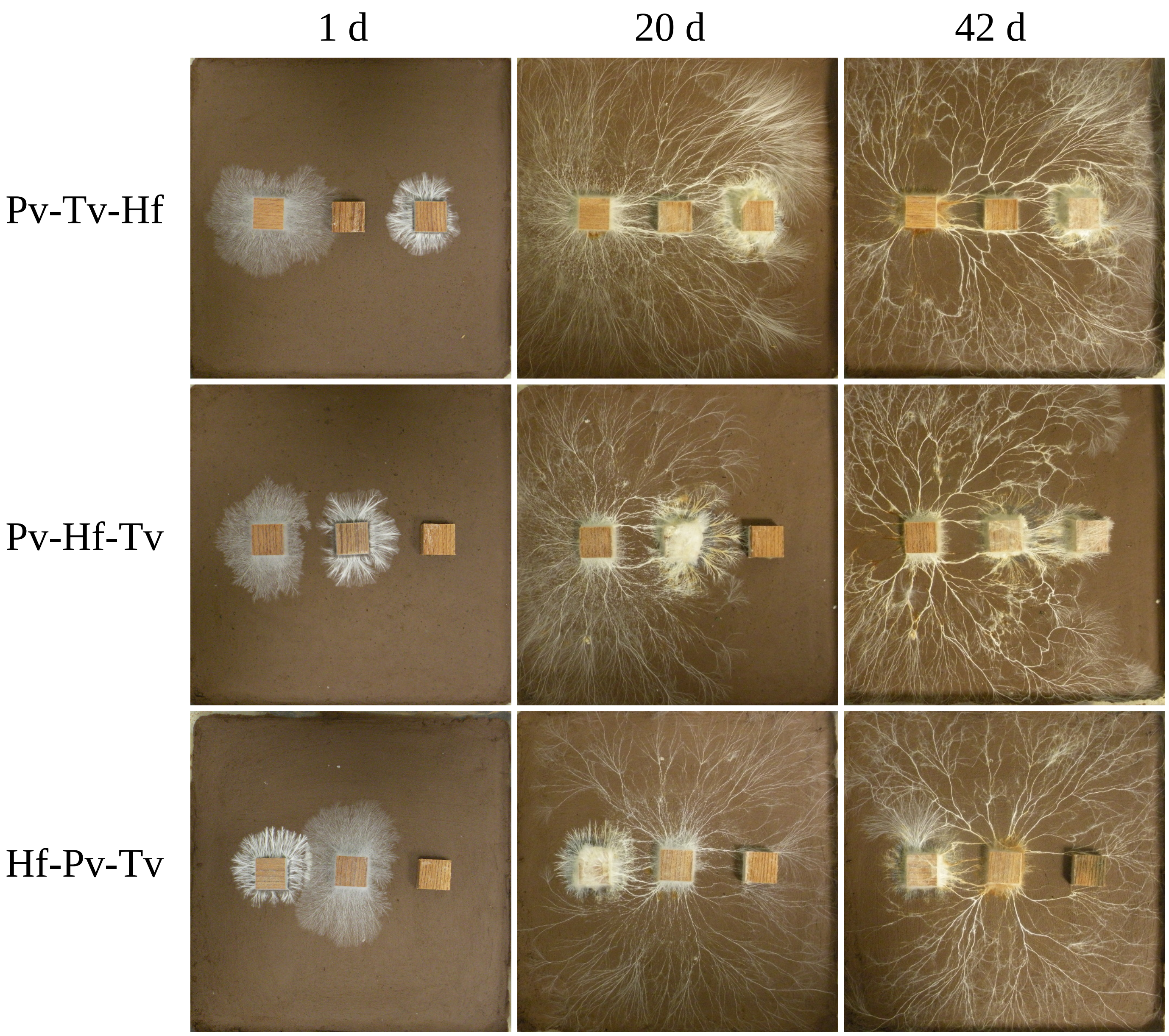

Supplementary Figure 1C. Progression of mycelial interactions between the cord-forming fungi *P. velutina* (Pv) and *H. fasciculare* (Hf) with the non-cord-forming fungus *T. versicolor* (Tv), growing out of pre-colonised beech wood blocks. Relative position of the blocks is assorted in all possible combinations.

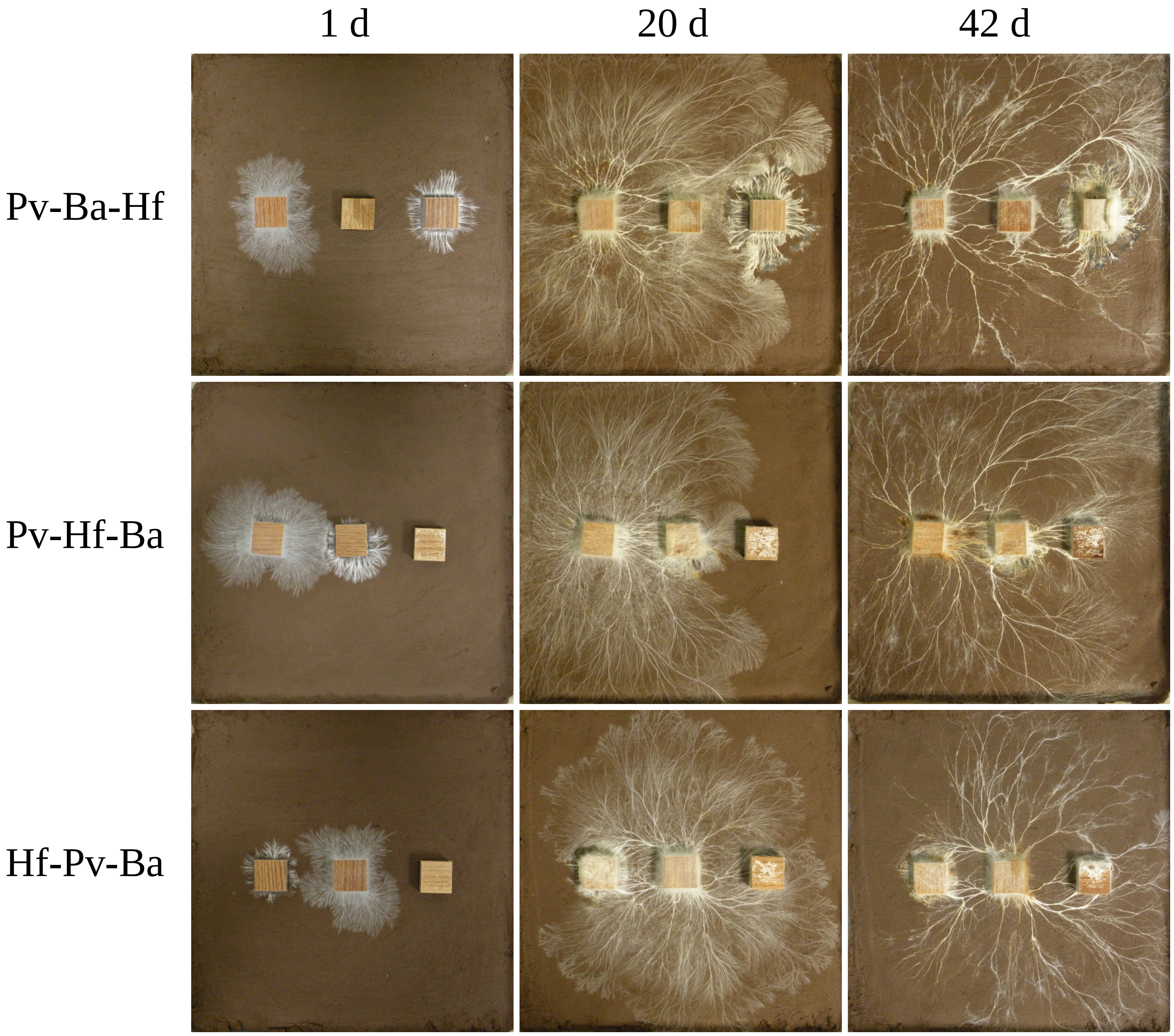

Supplementary Figure 1D. Progression of mycelial interactions between the cord-forming fungi *P. velutina* (Pv) and *H. fasciculare* (Hf) with the non-cord-forming fungus *B. adusta* (Ba), growing out of pre-colonised beech wood blocks. Relative position of the blocks is assorted in all possible combinations.

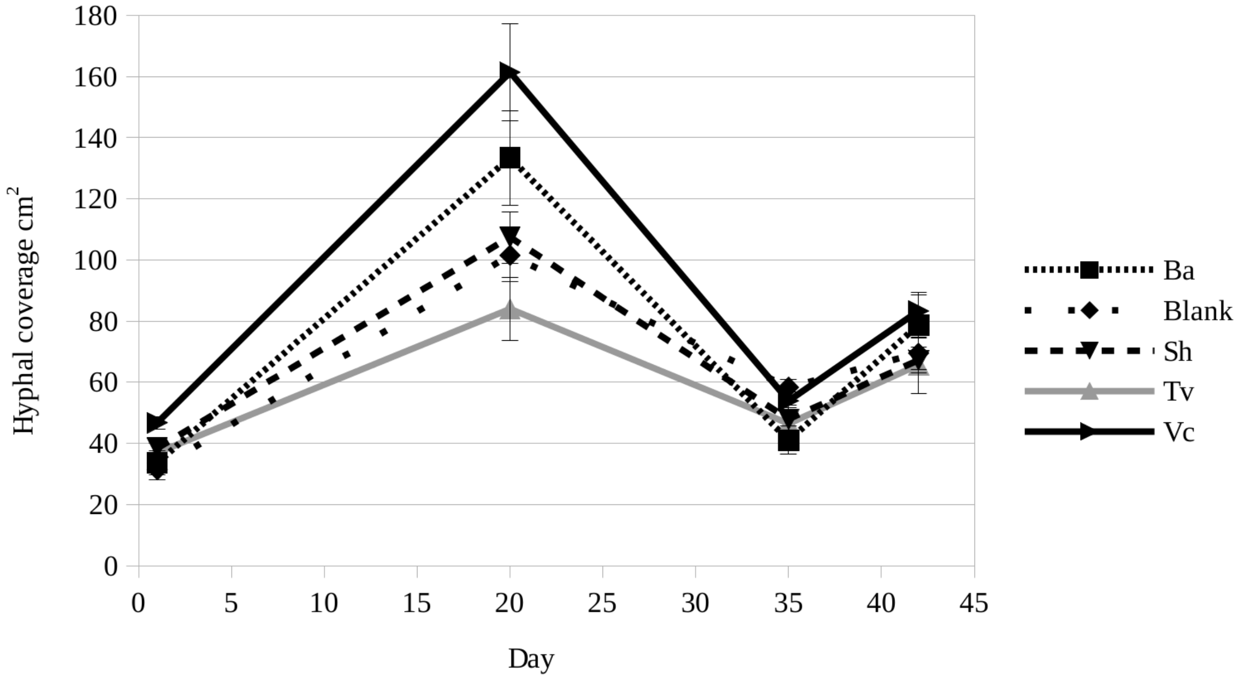

Figure S2. Changes in hyphal coverage of cord forming fungi during interactions with non-cord-forming fungi or uncolonised resources in soil trays. Hyphal coverage of cord formers over the time course of the interaction was measured at 1, 20, 35, and 42 d. Points indicate the mean hyphal coverage (n=7) ± standard error of the mean. Ba: *B. adusta*; Blank: uncolonised wood block; Tv: *T. versicolor*; Sh: *S. hirsutum*; Vc: *V. comedens*.

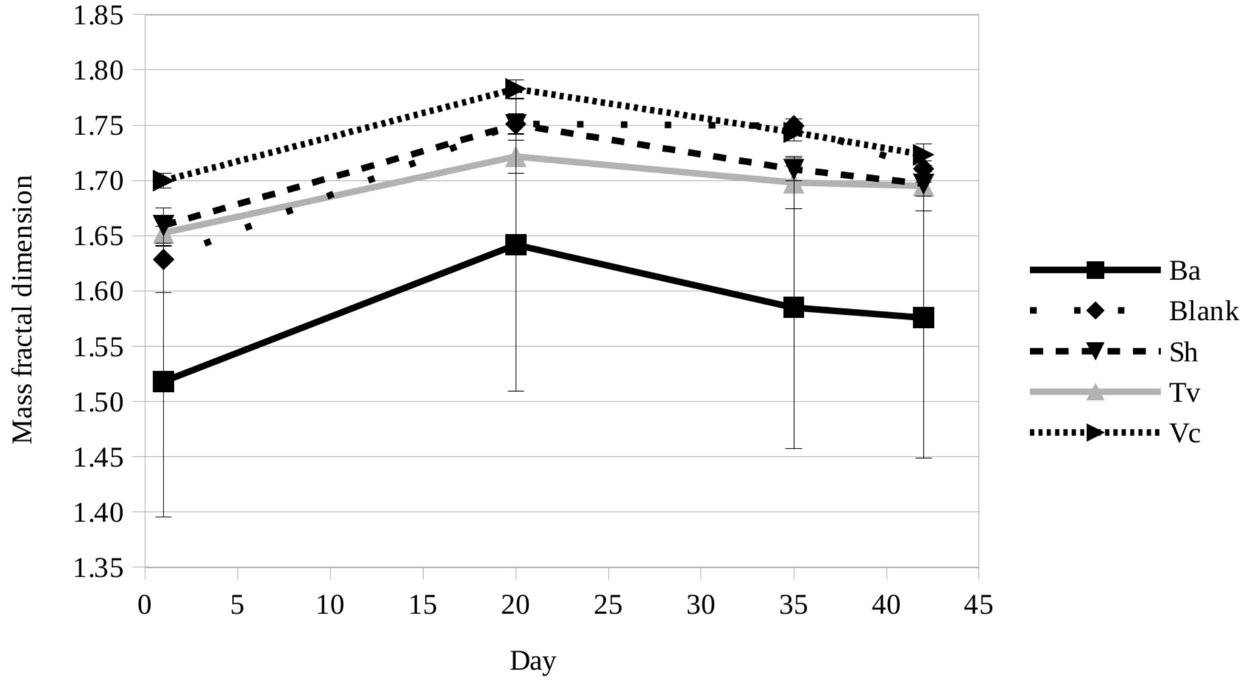

Figure S3. Changes in fractal dimension of cord forming fungi during interactions with non-cord-forming fungi or uncolonised resources in soil trays. Fractal dimension of cord formers over the time course of the interaction was measured at 1, 20, 35, and 42 d. Points indicate the mean fractal dimension (n=7) ± standard error of the mean. Ba: *B. adusta*; Blank: uncolonised wood block; Tv: *T. versicolor*; Sh: *S. hirsutum*; Vc: *V. comedens*.
